# Initial Conservative Management of Exomphalos Major with Gentian Violet

**Published:** 2012-10-01

**Authors:** Ashrarur Rahman Mitul, KMN Ferdous

**Affiliations:** Department of Pediatric Surgery, Dhaka Shishu (Children) Hospital Dhaka, Bangladesh.

**Keywords:** Exomphalos major, Conservative treatment, Gentian violet

## Abstract

Aim: The purpose of the study was to assess the results of topical use of gentian violet (GV), among the babies with exomphalos major in our institute.

Methods: The study was carried out retrospectively in a tertiary care hospital during the period from 2005 to 2010 inclusive. Exomphalos patients were classified as major if diameter was >5 cm and/or had liver in the sac as content. These patients were initially preferentially treated conservatively with topical 1% GV over the sac resultig a ventral hernia to be repaired later.

Results: A total of 84 exomphalos patients were admitted during the study period. Among them, 37 neonates (26 males and 11 females) had exomphalos major (EM). Ten of them were prenatally diagnosed. The mean gestational age at delivery was 35 weeks, and mean birth weight was 2.1 Kg. Mean age at presentation was 3.7 days. Thirty (81%) had other associated anomalies, mostly cardiac (66.6%) and pulmonary (46.6%). Ten patients with EM needed early operation because of ruptured sac, and other anomalies. There were 2 pre-operative and 8 postoperative deaths in this subgroup. Twenty seven patients were treated conservatively, among these 4 died of overwhelming sepsis. Remaining 23 patients left the hospital with a ventral hernia planned to be repaired at 1 year of age. Overall mortality in our series was 37.83%.

Conclusion: Initial conservative treatment of the sac with GV results in satisfactory outcome for infants with EM who cannot undergo immediate closure.

## INTRODUCTION

The incidence of exomphalos is approximately 1 in 3000 to 6000 live births [1, 2]. Some studies have classified this into 'minor' or 'major' (or alternatively 'giant') depending on the diameter of the abdominal wall defect (for instance, <5 and >5 cm or based on nature of contents (e.g. liver), whereas others have not discriminated [3]. Ein et al defined giant exomphalos as those having diameter of >10 cm [4]. Children with exomphalos major (EM) pose considerable problem. The most important issue is to choose between operative and non-operative treatment when simple closure is not possible in patients with EM. In the developing countries, management of babies with exomphalos remains a challenge to the pediatric surgeons because of lack of adequate neonatal intensive care facilities and total parenteral nutrition (TPN) [5]. Non-operative treatment using drying agents was first described by Ahlfeld in 1899 [6]. It still has its advocates. If managed successfully, the child is left with a soundly healed abdominal wall with a ventral hernia. The hernia can be repaired at an appropriate older age. A variety of agents have been used for epithelialisation (alcohol, mercurochrome, povidone iodine, gentian violet (GV), silver sulfadiazine, silver nitrate [2, 4-9]. In the index study, conservative treatment of EM with GV local application was preferred to operative management because of the lack of adequate neonatal intensive care facilities.

## MATERIAL AND METHODS

ondition, condition of the skin over the defect, any side effects attributable to GV. The repair of ventral hernia was undertaken at or after one year of age. All the repairs were possible as a single stage procedure. At surgery, the scar was incised via a midline incision, ventral hernia scar flaps were mobilized laterally with cautery until both the recti were either visualized or palpated. The liver was stuck to most of the upper hernia scar, was carefully identified and protected. If the lateral scar flaps along with the recti could be approximated without tension (n=7) to the midline, closure was accomplished. If the recti often could not be brought to the midline (n=4), a prolene mesh was used to complete the gap.

**Figure:**
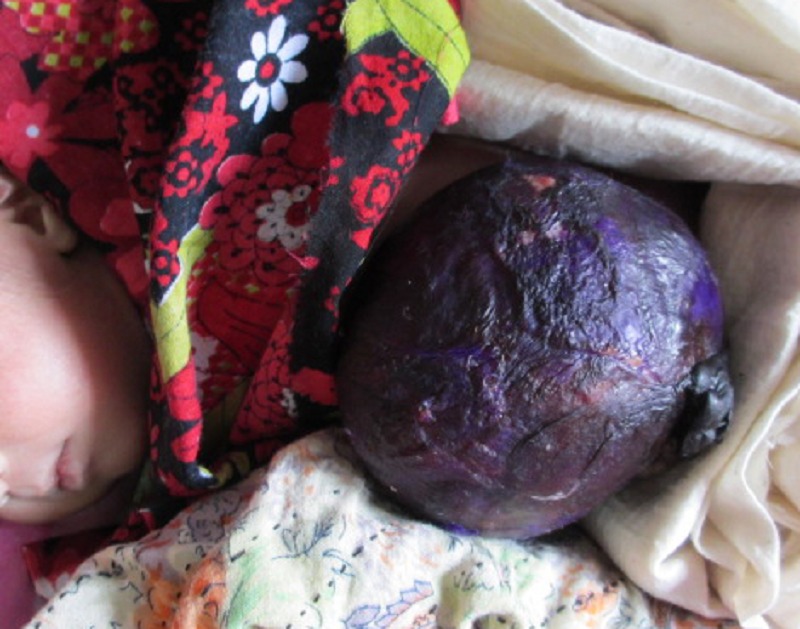
Figure 1: GV application over omphalocele.

## RESULTS

Eighty four neonates with exomphalos were admitted in our institute over the study period. Among them, we had 37 EM. The demographics of the study group are shown in Table 1. All (n=10) prenatally diagnosed patients were delivered by caesarian section (CS), 3 at term and 7 between 34 and 36 weeks. Among the remaining (n=27), 19 neonates underwent CS for obstetrical reasons; the rest (n=8) had vaginal delivery. Thirty (81%) patients had other associated anomalies, mostly cardiac (66.6%) and pulmonary (46.6%) (Table 2). Karyotyping was not available in this series. The majority of babies presenting late were septicemic, hypothermic, dehydrated, lethargic and reluctant to feed. Five babies with EM presented with ruptured sac and 3 had sac ruptured while on conservative treatment. Ten babies with EM needed early operation for sac rupture (n=8) or intestinal obstruction (n=2). Two patients who required surgery died during the preoperative period. Eight patients underwent surgery with silo formation (n= 5) or skin closure only (n=3). All of these babies died postoperatively because of respiratory and cardiac failure, silo separation, associated anomalies and/or overwhelming sepsis. Twenty seven babies were treated with GV were doing well, 4 of them succumbed in the 2nd week due to cardiac failure; echocardiographic diagnoses were Tetralogy of Fallot, ventricular septal defect, patent ductus arteriosus and pulmonary stenosis. Enteral feeding was started on 3rd to 7th postoperative day depending on the condition of the patient. Granulation tissue formation started by 3rd to 4th week and the gap started reducing in size. Epithelialisation and skin coverage usually completed by 4 to 6 weeks with formation of a ventral hernia (Fig. 2). Length of hospital stay was from 3 to 4 weeks. GV is cheap; total cost of 4 to 6 weeks treatment was only USD 2-3. Overall mortality was 37.83%. Survival of patients treated with GV was 85.18%.

**Figure:**
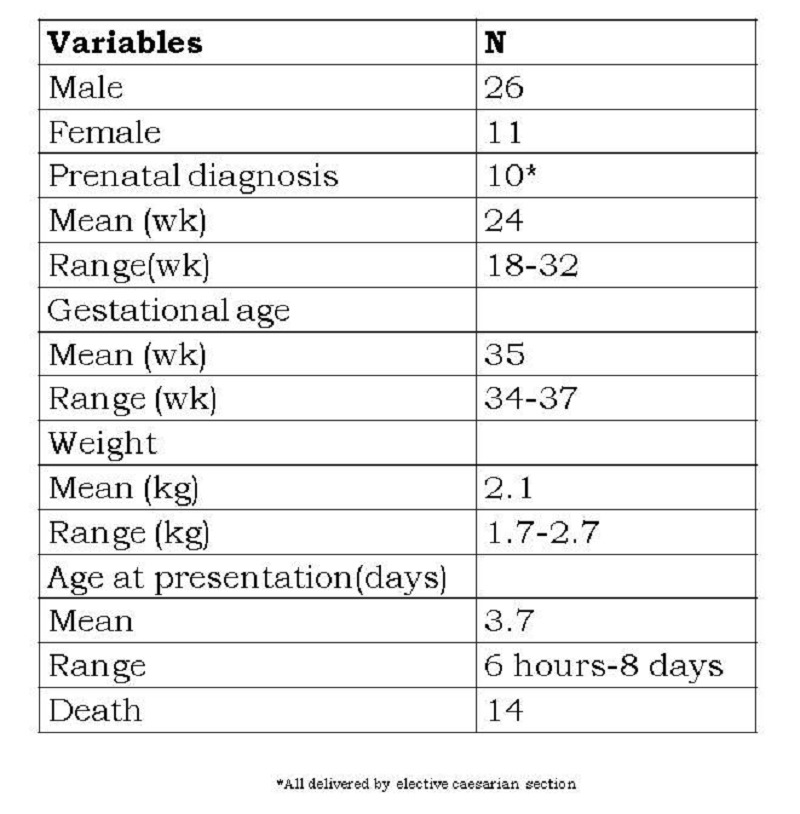
Table 1: Patient demographics (N=37)

**Figure:**
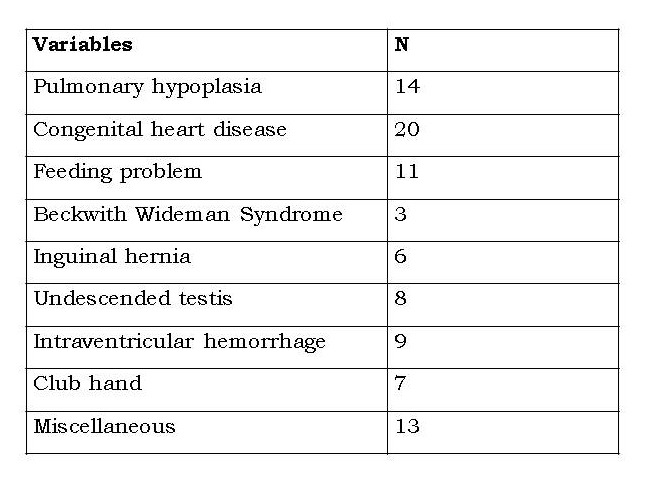
Table 2: Associated anomalies and/or medical problems (n=30)

**Figure:**
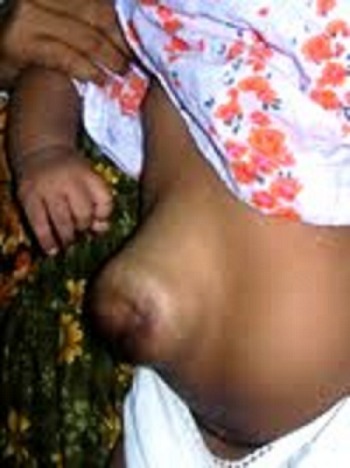
Figure 2: Defect epithelialised to form ventral hernia.

Follow up: Twenty patients with EM were under follow up for maximum of 18 months. There was satisfactory skin coverage over the ventral hernia; no adverse effect related to GV was noted. Eleven of them have already undergone ventral hernia repair with a satisfactory scar and no postoperative complications. Seven ventral hernias could be repaired without any prosthesis; rest 4 required prolene meshes. No prosthetic mesh related complication was observed till date. Remaining 9 patients are still living with the ventral hernia; repair of hernia is being delayed for other medical causes like poor general health and coexisting diseases. Three patients were lost to follow up.

## DISCUSSION

reign body leading to dehiscence, fistula formation, delay in enteral feeding, and high incidence of systemic sepsis [15]. We also encountered similar complications in our study with ultimate demise of the patients.

Our data suggests that non operative treatment leads to early enteral feeding and short hospital stay. Conservative treatment has the advantage of avoiding abdominal surgery in the neonatal period, and it averts the risk of tight abdominal closure as well as the particular complications of SC [15]. Nuchtern et al recommends that nonoperative treatment of large omphalocele is safer than SC and results in early enteral feeding and shorter hospital stay [15]. 

Conservative treatment, our mainstay of therapy, has been described by others too [5, 13]. A variety of agents have been used to paint the sac to promote eschar formation and help epithelialisation (alcohol, mercurochrome, povidone iodine, silver sulphadiazine, silver nitrate) [8, 13, 16]. But all of these agents are not free of risks. Mercury poisoning with mercurochrome, hypothyroidism with Povidone Iodine, silver toxicity with silver nitrate and silver sulphadiazine, alcohol toxicity have all been reported [5, 9, 17]. GV has been used by other authors as well [8, 9, 13]. GV is a triarylmethane antiseptic dye, has been in use since 1890. The name is due to its colour, it is not made from gentian or violet flowers. It has antibacterial, antifungal, and anthelmintic properties and has traditionally been used for treatment of several dermatological conditions, e.g., fungal infections( oral thrush, vaginal candidiasis), superficial bacterial infections like boils, chronic leg ulcers, methicilline resistant staph. aureus (MRSA), oral leukoplakia in HIV positive individuals. It is thought to work by inhibiting reactive oxygen species. Other uses include prevention of umbilical sepsis in newborns, control threadworms, to prevent blood transmission of Chaga's disease, as basis of Gram stain. It has some industrial uses as well. Side effects include irritation of mucous membrane at high concentration, oral ulceration, necrotic skin reaction at high concentration, staining of cloths. Studies have shown that GV is capable of causing cancer in mice [20], but there is no evidence of this occurring in humans. Serious side effects are rare. It is available as 0.5-2% solution [21]. Chan [7] and Mullin [8] have mentioned it to be safe for use in exomphalos, only disadvantage being that it stains linen. In the index study, we have used 1% GV as have others [7]. It is inexpensive; a total cost of 4 to 6 weeks treatment was 2-3 USD. We did not experience any adverse effects related to GV.

 Associated anomalies in babies with exomphalos are a major concern reported in several series ranging from 30% to 77% [13-15, 18]. In the present study associated anomalies were significantly high. We encountered significant number of cardiac and pulmonary anomalies. This is similar to the series reported by Lee et al [2] and Kumar et al [18] Overall mortality rate in author's study corroborated with those reported by other researchers which is between 30-50% [2-4]. Mortality in this study was related to sepsis, complications related to delayed presentation, associated anomalies, prematurity, respiratory and cardiac failure and other medical problems, inadequate intensive care facilities, and lack of total parenteral nutrition. This has been reflected in the report by several other authors [2, 4, 5, 19]. We encountered encouraging outcome with satisfactory survival rate, reduced LOS, early enteral feeding, shorter hospital stay with conservative treatment and no adverse effects with gentian violet. These results correlate with several other studies [2-4, 5, 8, 9, 13, 15].

## CONCLUSION

From the study in our institute, we can conclude, in resource challenged situations, conservative management of EM with delayed closure of the ventral hernia should be the choice of management. Associated anomalies and other medical problems should be addressed as well at the same time for better out come. A multidisciplinary approach involving the neonatologists, intensivist and neonatal surgeons may be more rewarding. Gentian violet is an inexpensive and safe escharotic agent, is effective in the treatment. It can be a better alternative to other agents with proven toxicity.

## Footnotes

**Source of Support:** Nil

**Conflict of Interest:** None declared
